# Role of Macrophages in the Altered Epithelial Function during a Type 2 Immune Response Induced by Enteric Nematode Infection

**DOI:** 10.1371/journal.pone.0084763

**Published:** 2014-01-23

**Authors:** Luigi Notari, Diana C. Riera, Rex Sun, Jennifer A. Bohl, Leon P. McLean, Kathleen B. Madden, Nico van Rooijen, Tim Vanuytsel, Joseph F. Urban, Aiping Zhao, Terez Shea-Donohue

**Affiliations:** 1 Department of Medicine and Mucosal Biology Research Center, University of Maryland School of Medicine, Baltimore, Maryland, United States of America; 2 Department of Pediatrics, Walter Reed Army Medical Center, Washington, DC, United States of America; 3 Department of Pediatrics, Uniformed Services University of the Health Sciences, Bethesda, Maryland, United States of America; 4 Vrije Universiteit, VUMC, Department of Molecular Cell Biology, Amsterdam, The Netherlands; 5 Translational Research Center for Gastrointestinal Disorders, University Hospital Gasthuisberg, University of Leuven, Leuven, Belgium; 6 United States Department of Agriculture, Agricultural Research Service, Beltsville Human Nutrition Research Center, Diet, Genomics, & Immunology Laboratory, Beltsville, Maryland, United States of America; The Hospital for Sick Children and The University of Toronto, Canada

## Abstract

Parasitic enteric nematodes induce a type 2 immune response characterized by increased production of Th2 cytokines, IL-4 and IL-13, and recruitment of alternatively activated macrophages (M2) to the site of infection. Nematode infection is associated with changes in epithelial permeability and inhibition of sodium-linked glucose absorption, but the role of M2 in these effects is unknown. Clodronate-containing liposomes were administered prior to and during nematode infection to deplete macrophages and prevent the development of M2 in response to infection with *Nippostrongylus brasiliensis*. The inhibition of epithelial glucose absorption that is associated with nematode infection involved a macrophage-dependent reduction in SGLT1 activity, with no change in receptor expression, and a macrophage-independent down-regulation of GLUT2 expression. The reduced transport of glucose into the enterocyte is compensated partially by an up-regulation of the constitutive GLUT1 transporter consistent with stress-induced activation of HIF-1α. Thus, nematode infection results in a “lean” epithelial phenotype that features decreased SGLT1 activity, decreased expression of GLUT2 and an emergent dependence on GLUT1 for glucose uptake into the enterocyte. Macrophages do not play a role in enteric nematode infection-induced changes in epithelial barrier function. There is a greater contribution, however, of paracellular absorption of glucose to supply the energy demands of host resistance. These data provide further evidence of the ability of macrophages to alter glucose metabolism of neighboring cells.

## Introduction

Enteric nematodes co-evolved with the mammalian immune system and developed extremely effective strategies to modulate and evade host defenses and maintain their evolutionary fitness. The host response to nematode infection is characterized by induction of a T-helper 2 (Th2) immune response, involving the elevated production of inteleukin-4 (IL-4), IL-5, IL-10 and IL-13 [1]. This response is coincident with recruitment of specific immune cells, such as mast cells, eosinophils, basophils, and macrophages, to the infection site [2]. There growing recognition of the inverse correlation between the increased incidence of autoimmune diseases, including diabetes, and the decreased incidence enteric infections, including nematodes. There is evidence that nematode infection inhibits the development of type-1 diabetes in non-obese diabetic (NOD) mice [3], [4] and attenuates the metabolic syndrome induced by a high fat diet [5]. The mechanisms involved in the protective effect of enteric nematode parasites remain an active area of investigation with regard to their therapeutic potential.

Two major functions of the intestinal epithelium are to act as a physical barrier between the external and internal milieu and to regulate the movement of nutrients, ions, and fluid. Nematode infection induces stereotypic alterations in smooth muscle and epithelial cell function that facilitate worm expulsion [6]–[8]. Increased fluid within the lumen is attributed to a STAT6-dependent increase in intestinal permeability and inhibition of sodium-linked glucose absorption [7]. Glucose absorption in intestinal cells occurs at the brush border membrane primarily through the unidirectional sodium-dependent glucose transporter-1 (SGLT-1). Other transporters, GLUT2 and GLUT5, drive facilitated transport of carbohydrates in the small intestine and complement SGLT1 function [9]. GLUT5 is primarily expressed in the brush border membrane and exclusively transports fructose. GLUT2 is expressed primarily in the basolateral membrane and provides a common pathway for glucose and fructose transport out of the epithelial cell [10]–[12]. The constitutively expressed non-insulin dependent transporter GLUT1 does not normally contribute to glucose transport in murine small intestinal enterocytes, but recent studies demonstrated expression of GLUT1 on the basal aspect of colonocytes [13]. Clinical diabetes has been linked to increased expression of SGLT1 and GLUT2 and these transporters are considered to be therapeutic targets in diabetes [14]. Earlier studies in *N. brasilinesis*-infected rats showed decreased expression of SGLT1 and increased expression of GLUT1 limited to the site of infection [15], although the mechanisms and functional implications of these changes remain unclear.

The intestinal lamina propria contains the largest resident population of macrophages in the body and these cells play a key role in immune homeostasis [16]. The number of macrophages within the lamina propria increases in response to nematode infection or inflammation and the resulting macrophage phenotype and function are dependent on the cytokine environment and site of inflammation [17]. The presence of Th1 cytokines such as interferon-γ (IFN-γ) promotes development of pro-inflammatory classically activated macrophages (M1) [18]. In contrast, up-regulation of the Th2 cytokines, IL-4 and IL-13, during nematode infection and allergy leads to development of alternatively activated macrophages (M2) that are important for tissue repair. These macrophage phenotypes can be distinguished by up-regulation of different surface markers and enzymes including inducible nitric oxide synthase (NOS-2) or arginase-1, which both metabolize L-arginine, respectively, to nitric oxide (M1) or to proline and polyamines (M2) [17].

Pro-inflammatory M1 infiltrate adipose tissue in obesity [19], [20] and play a critical role in the development of insulin resistance [21]. In contrast, the presence of M2 in adipose tissues is linked to protection against obesity and insulin resistance (reviewed in [22]). Indeed, adipose macrophages from healthy mice resemble M2 and are necessary for the maintainenance of glucose homeostasis [23]. In addition, there is evidence that preventing the development of M2 renders mice susceptible to diet-induced obesity and glucose intolerance [24], [25]. Finally, our recent data show that nematode infection improves oral glucose tolerance tests in high fat diet induced obesity, an effect that was attributed to the presence of M2 in epididymal fat [5]. These data suggest that macrophages alter function in neighboring cells and may contribute to the nematode-infection induced alterations in glucose absorption of enterocytes in the small intestine.

The current study was designed to investigate the role of macrophages in nematode-induced alterations in epithelial cell function and glucose absorption. The results of our studies demonstrate that nematode infection alters intestinal glucose absorption by macrophage-dependent and -independent mechanisms. The increased intestinal permeability and luminal fluid accumulation activated by parasitic nematode-induced IL-4/IL-13 production results in relatively greater paracellular absorption of glucose. We conclude that nematode infection results in a “lean” epithelial cell phenotype, in reference to the low intracellular glucose as a result of reduced SGLT1 activity and down-regulation of GLUT2 and GLUT5 expression. This leads to an up-regulation of GLUT1 expression that serves as the major compensatory mechanism for glucose entry into the enterocyte.

## Materials and Methods

### Mice

Wild type (WT) age-matched female BALB/c mice were purchased from the Small Animal Division of the National Cancer Institute. Female mice deficient in STAT6 (STAT6^−/−^), on a BALB/c background, were obtained from Jackson Labs. Animals were euthanized using ketamine/xylazine prior to the collection and processing of tissues. All studies were conducted in accordance with the principles set forth in the Guide for Care and Use of Laboratory Animals, Institute of Laboratory Animal Resources, National Research Council, Health and Human Services Publication (National Institutes of Health 85-23, revised 1996), and the Beltsville Area Animal Care and Use Committee, protocol #10-003. The protocol was also approved by the Institutional Animal Care and Use Committee of the University of Maryland School of Medicine (Protocol number 0110018).

### *Nippostrongylus brasiliensis* infection and worm expulsion

Infective, third stage larvae (L_3_) of *Nippostrongylus brasiliensis* (*N. brasiliensis*) (specimens on file at the U.S. National Parasite Collection, U.S. National Helminthological Collection, Collection 81930, Beltsville, MD) were propagated and stored at room temperature in fecal/charcoal/peat moss culture plates until used [8]. Groups of mice were inoculated subcutaneously with 500 L_3_ and studied nine days later. We showed previously that the maximal elevation of Th2 cytokines occurs at day 7 post-inoculation with *N. brasiliensis* L3 and precedes the changes in gut function at days 8–9 that coincide with worm expulsion [8]. Appropriate age-matched controls were studied concurrently.

### Liposome-mediated macrophage depletion

*In vivo* macrophage depletion was performed as described previously [26]. Briefly, groups (n = 5) of WT or STAT6^−/−^ mice were inoculated with *N. brasiliensis* L3 or treated with vehicle. To deplete macrophages, mice received clodronate- (Cl_2_MDP) or control PBS-containing liposomes (0.2 ml, *i.v.*) daily, beginning one day before and continuing for nine days post inoculation. Liposomes were generated as described previously using phosphatidylcholine (LIPOID E PC; Lipoid GmbH, Ludwigshafen, Germany) and cholesterol (Sigma, St. Louis, MO) [27], [28]. Mice were euthanized and body weight recorded after nine days of daily injections.

#### RNA extraction, cDNA synthesis and quantitative real-time polymerase chain reaction (qRT-PCR)

As per manufacturer's instructions, total RNA was extracted from full thickness sections of small intestine with TRIzol reagent (Invitrogen, Carlsbad, CA). RNA integrity, quantity, and genomic DNA contamination were assessed using the Agilent Bioanalyzer 2100 and RNA 6000 Labchip kit (Agilent Technologies, Palo Alto, CA). Only those RNA samples with 28S/18S ratios between 1.5 and 2 and no DNA contamination were studied further. RNA samples (2 µg) were reverse-transcribed to cDNA using the First Strand cDNA Synthesis Kit (MBI Fermentas, Hanover, MD) with random hexamer primer.

Amplification reactions were performed on an iCycler detection system (Bio-Rad, CA). Primer sequences were designed using Beacon Designer 5.0 (Premier Biosoft International, Palo Alto, CA), and synthesized by the Biopolymer Laboratory of the University of Maryland. The primer sequences that were not described previously by our lab [29], [30] including those for SGLT1, GLUT1, GLUT2, and GLUT5 are listed on Table 1. PCR was performed in 25 µl volume wells using SYBR green Supermix (Bio-Rad, Hercules, CA). Amplification conditions were: 95°C for 3 min, 60 cycles of 95°C for 15 s, 60°C for 15 s, and 72°C for 20 s. The fold-change in mRNA expression for targeted genes was calculated relative to the respective vehicle-treated groups of mice after normalization to 18 s rRNA. We selected 18 s rRNA for the internal standard in our qRT-PCR, based on our preliminary studies showing that there were no significant differences in the 18 s rRNA level among the different groups of samples (infected and uninfected).

**Table 1:** Sequences of the sense and antisense primers used for quantitative real time RT-PCR.

Gene	Sense Primer	Antisense Primer
**Arg1**	GAGTATGACGTGAGAGAC	TTCTTCACAATTTGAAAGGA
**CD206**	TTTGGAATCAAGGGCACAGAG	TGCTCCACAATCCCGAACC
**Fizz1**	CCTCCACTGTAACGAAGACTCTC	GCAAAGCCACAAGCACACC
**F4/80**	AAAGACTGGATTCTGGGAAGTTTGG	CGAGAGTGTTGTGGCAGGTTG
**NOS2**	CGGAGCCTTTAGACCTCAACA	CCCTCGAAGGTGAGCTGAAC
**SGLT1**	CATCAGCGTCATCACCATCTTG	GTCCAGCCCACAGAACAGG
**Glut1**	CTGCTCAGTGTCGTCTTC	ACCCTCTTCTTTCATCTCC
**Glut2**	ACAGTCACACCAGCATAC	ACCCACCAAAGAATGAGG
**Glut5**	CAGGTATCTCTTCCAACG	CCATCCTCATTTGCCAAG
**HIF-1a**	ATGAGAGAAATGCTTACACAC	TGAGGTTGGTTACTGTTGG
**Relm-β**	TCTCCCTTTTCCCACTGATAG	TCTTAGGCTCTTGACGACTG

#### Ussing Chambers

One centimeter segments of jejunal mucosa were stripped of *muscularis externa* and mounted in Ussing chambers exposing 0.126 cm^2^ of the epithelium to 10 ml of Krebs' buffer. Potential difference was measured using agar-salt bridges and electrodes. Every 50 s, the tissue was short circuited at 1 V (World Precision Instruments DVC 1000 voltage clamp, Sarasota, FL), and the short circuit current (I_sc_) was monitored continuously. Also, every 50 s, the clamp voltage was adjusted to 1 V for 2 s to allow calculation of tissue resistance utilizing Ohm's law (V = IR). Basal I_sc_, representing the net ion flux at baseline, and tissue resistance, an indication of tissue permeability, were determined after a 15 min period of equilibrium. Following a second 15 min period, concentration-dependent changes in I_sc_ were measured in response to the cumulative addition of glucose to the mucosal side. This is a measurement of sodium-linked glucose transport that can be attributed directly to the activity of the SGLT1. For all tissue segments taken from an individual mouse (n = 3–4 per mouse), resistance, basal I_sc_, and changes in I_sc_ in response to glucose were averaged to yield a mean response per animal and then averaged to yield a mean value per group.

### Preparation of frozen tissue blocks and cryo-sectioning

Tissues were taken from the jejunum of *N. brasiliensis*-infected mice, slit longitudinally, prepared using the Swiss-roll technique [31], [32], embedded in Tissue-Tek OCT compound (Sakura Finetek U.S.A., Torrance, CA), frozen on dry ice-acetone, and stored at −80°C.

### Laser capture micro-dissection

For laser capture micro-dissection (LCM), 4-µm tissue sections were cut from frozen tissue sections of a Swiss-roll preparation using an HM505E cryostat (Richard-Allan Scientific, Kalamazoo, MI). The sections were dehydrated and stained for H&E (Sigma-Aldrich, St. Louis, MO). LCM of stained sections was performed on a PixCell II (Arcturus Engineering, Mountain View, CA), and captured cells were transferred to CapSure™ LCM Caps (Arcturus Engineering, Mountain View, CA). The LCM cap was inserted into a 0.5-ml micro-centrifuge tube containing RNA isolation solution, and total RNA was extracted using an RNA isolation kit (Stratagene Cloning Systems, La Jolla, CA). Cells were captured from the region of the epithelium and lamina propria in the small intestine as previously described [32], [33].

### Western blotting

Protein samples were resolved using Novex 4–12% polyacrylamide gels with Tris glycine-SDS running buffer (Invitrogen, Grand Island, NY). After electrophoresis proteins from the gel were transferred onto a nitrocellulose membrane (Invitrogen, Grand Island, NY). The membranes were blocked in 5% non-fat milk/Tris buffered saline plus 0.1% Tween-20, (TBS-T) for 1 h at room temperature. Primary antibodies were goat anti-SGLT1 diluted at 1∶2,000 (Santa Cruz Biotechnology, Cat# sc-20582), goat anti-Glut2 diluted at 1∶2,000 (Santa Cruz Biotechnology, Cat# sc-31835), rabbit anti-GLUT1, diluted at 1∶500 (Abcam, Cat# ab32551), mouse anti β-actin, diluted 1∶2,000 (Thermo Scientific, Cat# 82353) in 5% non-fat milk/TBS-T. Secondary antibodies were HRP-conjugated donkey anti goat IgG, goat anti rabbit IgG, or goat anti-mouse IgG (KPL, Gaithersburg, MD), diluted at 1∶10,000 in 5% non-fat milk/TBS-T. For detection, membranes were incubated in SuperSignal West Pico Chemiluminescent Substrate (Thermo Scientific, Cat# 34080) and chemiluminescent bands were acquired using a Fuji Film Intelligent Dark Box with LAS-4000 software version 2.1.

### Immunofluorescence

Mouse intestines were opened longitudinally along the mesenteric border, fixed for two hours in 4% paraformaldehyde, and embedded in paraffin blocks. For immunofluorescence, 5 µm sections on glass slides were de-waxed in xylene and rehydrated in descending ethanol baths. Antigen retrieval was achieved by incubating slides in sodium citrate buffer at 120°C for 10 minutes. Nonspecific binding of antibodies was blocked by incubation with 10% normal goat serum, 0.1% Triton X-100 in PBS (GLUT2) or 5% BSA, 0.1% Triton X-100 in PBS (SGLT1) for 1 hour at 25°C. Sections were incubated with 1∶200 dilutions of rabbit anti-mouse GLUT2 (Chemicon International) or 1∶100 dilutions of goat anti-mouse SGLT1 (Chemicon International) overnight at 4°C. After washing with PBS three times for 5 minutes, slides were incubated with TRITC-labeled anti-rabbit (GLUT2) or FITC-labeled anti-goat (SGLT1) antibodies and then washed with PBS three times for 5 minutes. Slides were mounted using Mounting Medium containing DAPI (Sigma, St. Louis, MO) and images were obtained using an Olympus microscope and Fluo View confocal software (Olympus America Inc., Central Valley, PA).

### Solutions and drugs

All drugs used for physiological studies were obtained from Sigma (St. Louis, MO) unless otherwise indicated.

### Data analysis

For multiple comparisons, statistical analyses were performed using a one-way analysis of variance (ANOVA) with post hoc analysis for multiple comparisons. Data analyses were performed using Graph Pad Prism software and differences between groups were considered statistically significant at p values<0.05.

## Results

### Macrophage markers during nematode infection

There was no significant difference in body weight among the four treatment groups (Table 2). We confirmed previous observations from our lab and others, showing that treatment with Cl_2_MDP prevented infection-induced infiltration of macrophages [26], [30]. The expression of F4/80 (a pan-macrophage marker) in full thickness sections of intestinal tissue was significantly elevated during infection indicating recruitment of macrophages (Table 2). Furthermore, specific markers for M2 such as arginase-1, mannose receptor (CD206) and FIZZ1 (found in areas of inflammation) were up-regulated significantly in full thickness sections of intestinal tissue during infection, when compared to controls (Table 2). In contrast, the marker for the classically activated pathway, NOS-2, was unaltered by infection. Treatment with Cl_2_MDP significantly attenuated the expression of F4/80 (not shown) as well as the M2 markers, with the exception of FIZZ1 (Table 2). We showed previously that expression of CD206 and arginase-1, but not FIZZ1, is dependent on STAT6 [30].

**Table 2:** Effect of PBS- or clodronate-containing liposomes on body weight (in grams) and gene expression (fold change) of M1 and M2 macrophage markers in response to *N. brasiliensis* infection.

			*N. brasiliensis* +	*N. brasiliensis* +
Parameter	PBS	Clodronate	+PBS	Clodronate
**Body Weight**	19.5±0.9	18.1±0.9	17.8±0.4	16.8±0.5
**F4/80**	1.0±0.1	0.22±0.05	2.70±0.4	0.24±0.02
**CD206**	1.0±0.5	0.3±0.05*	3.6±0.5*	0.52±0.06φ
**Fizz1**	1.0±0.3	0.58±0.2*	74±8.5*	32.0±10.9φ
**Arginase-1**	1.0±0.2	0.45±0.1*	10.10±0.9*	1.6±0.5φ
**NOS2**	1.00±0.78	ND	0.15±0.04	ND

Pan-macrophage marker F4/80, M2 markers arginase-1, Fizz1, and CD206, and a M1 marker NOS2.

* p<0.05 vs Control;

φ p<0.05 vs Clodronate;

ND = not detected.

### Effects of macrophage depletion on epithelial resistance and glucose absorption

**Figure 1:**
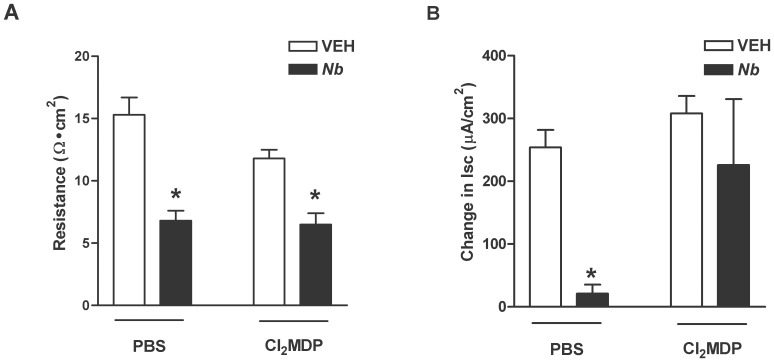
Macrophage depletion improves the *N. brasiliensis*-induced decrease in glucose absorption, but not in epithelial resistance. WT mice were given PBS or clodronate (Cl_2_MDP) containing liposomes to deplete macrophages and one day later treated with vehicle (VEH) or infected with *N. brasiliens*is (Nb). At day nine post inoculation, muscle-free mucosae were mounted in Ussing chambers to determine (A) mucosal resistance and (B) concentration dependent changes in epithelial sodium-linked glucose absorption. Values shown are means ± SE; *p<0.05 vs VEH Control.

Previous data from our laboratory showed that *N. brasiliensis* infection induced a decrease in mucosal resistance [7]. This increased mucosal permeability associated with decreased resistance during infection was observed also in *N. brasiliensis*-infected mice treated with Cl_2_MDP (Figure 1A) indicating that macrophages do not contribute to infection-induced changes in barrier function. In addition, we confirmed the observation that nematode infection decreased glucose absorption in Ussing chambers, consistent with reduced transport of glucose via the sodium-dependent glucose transporter SGLT1. In contrast to WT infected mice receiving PBS containing liposomes, glucose absorption in *N. brasiliensis*-infected mice treated with Cl_2_MDP (Figure 1B) was similar to that in uninfected mice. These data demonstrate that macrophages have a key role in regulating the changes in SGLT1-dependent glucose absorption induced by nematode infection.

### Effect of *N. brasiliensis* infection on the expression of glucose transporters

**Figure 2:**
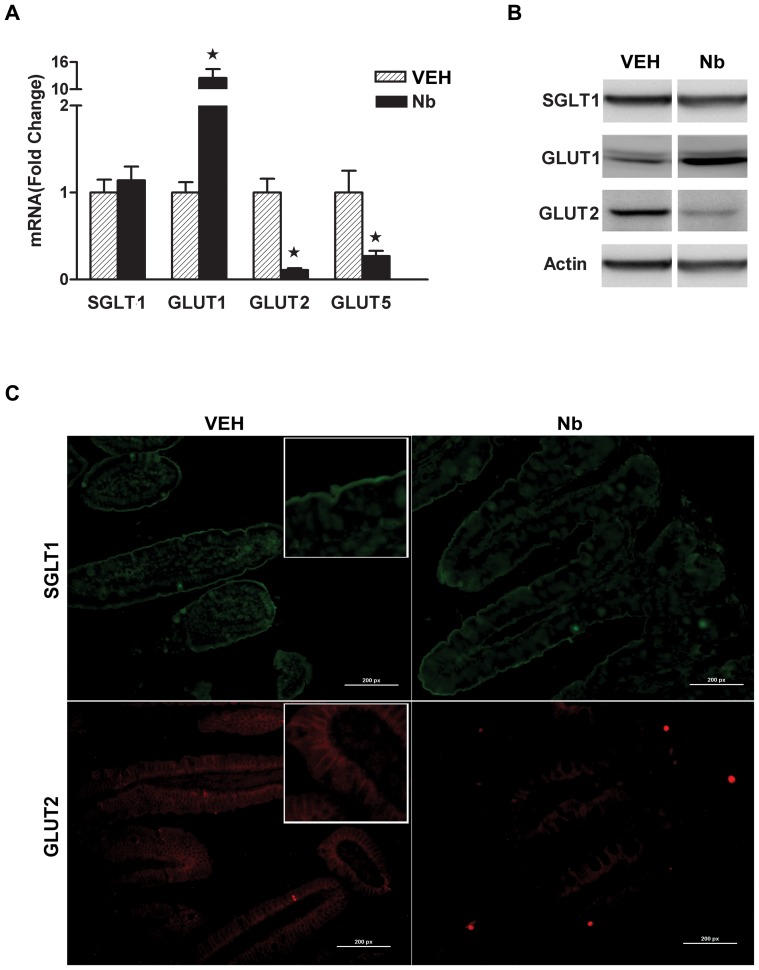
*Nippostrongylus. brasiliensis* infection alters the expression of glucose transporters. WT mice were treated with vehicle (VEH) or were infected with *N. brasiliensis* (Nb). At day nine post inoculation, full thickness sections of small intestine were prepared for (A) real-time qPCR to measure the mRNA expression of glucose transporters (B) Western blot analyses for glucose transporters, using actin as a loading control and (C) immunofluorescence staining for glucose transporters on the surface of villi of small intestine. The photomicrographs are representative of five different replicate slides. Values shown are means ± SE; p<0.05 vs VEH Control.

Given that Cl_2_MDP-mediated depletion of macrophages attenuated the decrease in sodium linked glucose absorption during nematode infection, we next investigated the effect of nematode infection on protein expression of glucose transporters in full thickness sections of small intestine. When compared to uninfected controls, there was no change in the mRNA or protein expression of SGLT1 induced by *N. brasiliensis* infection (Figure 2A and B). Visualization of immunofluorescence staining further confirmed that apical SGLT1 expression in mouse enterocytes was unaffected by *N. brasiliensis* infection (Figure 2C). These data demonstrate further that inhibition of sodium-linked glucose absorption during infection (Figure 1B) was due to decreased activity, rather than decreased expression, of SGLT1. There was no change in the expression of GLUT4 (data not shown); however, GLUT2 gene and protein expression were down-regulated significantly during *N. brasiliensis* infection when compared to uninfected controls (Figure 2A and B). Notably, immunofluorescence staining showed a marked reduction in the basolateral expression of GLUT2 at day 9 post inoculation (Figure 2C). In contrast, there was a significant up-regulation of GLUT1 gene and protein expression (Figure 2A and B).

### Expression of glucose transporters during *N. brasiliensis* infection in macrophage depleted mice

**Figure 3:**
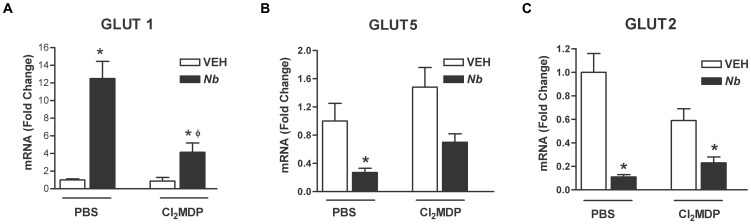
The effect of macrophage depletion on *N. brasiliensis* infection-induced changes in the expression of glucose transporters. WT mice were given PBS or clodronate (Cl_2_MDP) containing liposomes to deplete macrophages and one day later treated with vehicle (VEH) or infected with *N. brasiliens*is (Nb). At day nine post inoculation, full thickness sections of small intestine were prepared for real-time qPCR to measure the mRNA expression of glucose transporters. Values shown are means ± SE; *p<0.05 vs VEH Control; φp<0.05 vs *N. brasiliensis* infected mice treated with PBS-containing liposomes.

Macrophages express receptors for glucose including GLUT1 and GLUT5; therefore, we assessed the effects of macrophage depletion on the expression of these transporters. Changes in expression of GLUT1 were dependent, in part, on the presence of macrophages (Figure 3A) in that GLUT1 gene expression was reduced significantly in full thickness sections of small intestine taken from Cl_2_MDP-treated mice when compared to the mice given PBS liposomes, but also remained significantly elevated above levels in vehicle-treated group. The gene expression of GLUT5 in *N. brasiliensis*-infected mice treated with Cl_2_MDP was no longer different from either uninfected or infected mice (Figure 3B). In contrast, the infection-induced decrease in GLUT2 mRNA expression remained low even after treatment with Cl_2_MDP (Figure 3C), demonstrating that macrophages do not contribute to infection-induced changes in expression of GLUT2.

### Immune regulation of GLUT1 and GLUT2 gene expression

**Figure 4:**
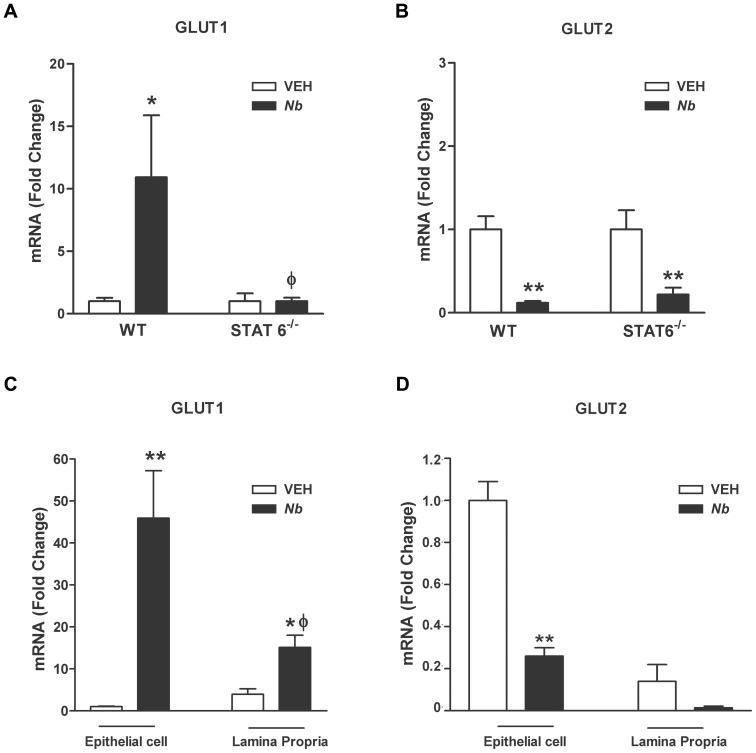
Immune regulation of glucose transporter expression and localization of GLUT1 and GLUT2 transporters. WT or STAT6^−/−^ mice were treated with vehicle (VEH) or were infected with *N. brasiliensis* (Nb). At day nine post infection, full thickness sections of small intestine were prepared for real-time qPCR to determine the mRNA expression of (A) GLUT1 or (B) GLUT2. WT mice were treated with vehicle (VEH) or were infected with *N. brasiliensis* (Nb). At day nine post inoculation, sections of small intestine were prepared for laser capture micro-dissection (LCM) to determine (C) GLUT1 or (D) GLUT2 mRNA expression in epithelial cells or in the lamina propria. Values shown are means ± SE; **p<0.05 vs VEH Control; φp<0.05 vs WT *N. brasiliensis*.

We showed previously that the nematode-infection inhibition of sodium-linked glucose transport through SGLT1 was STAT6-dependent [7], so we next determined the STAT6-dependence of infection-induced changes in GLUT1 and GLUT2 expression. GLUT1 and GLUT2 mRNA expression were measured in full thickness sections of small intestinal tissue from WT and STAT6^−/−^ mice infected with *N. brasiliensis* or treated with vehicle alone. The infection-induced increase in GLUT1 mRNA expression was attenuated in infected STAT6^−/−^ mice (Figure 4A), indicating that the infection-induced increase in GLUT1 mRNA is dependent on the STAT6 pathway and immune regulation. In contrast, GLUT2 changes in mRNA expression remained low in the infected STAT6^−/−^ mice (Figure 4B) confirming that these changes were not contingent on nematode-induced IL-4/IL-13 activation of STAT6.

### Localization of GLUT1 and GLUT2 via laser capture microdisection (LCM)

To further evaluate cell-specific changes in the expression of glucose transporters, we used LCM to isolate epithelial and lamina propria cells, followed by qRT-PCR. Surprisingly, GLUT1 mRNA was up-regulated significantly (Figure 4C) by *N. brasiliensis* infection in the epithelial cell layer when compared to uninfected controls. It was also increased in the lamina propria, the site of M2 recruitment, but not to the same level as in the epithelial layer (Figure 4C). These results demonstrate that in addition to macrophages, epithelial cells also up-regulate GLUT1 expression during nematode infection. The mRNA expression of GLUT2 was significantly decreased in the LCM-isolated epithelial cells during infection when compared to uninfected control (Figure 4D) confirming the results obtained with whole tissue. There very low expression of GLUT2 in the lamina propria (Figure 4D) is consistent with location of this receptor in the small intestine almost exclusively on epithelial cells.

### Mechanisms of regulation of epithelial glucose transporters in nematode infection

**Figure 5:**
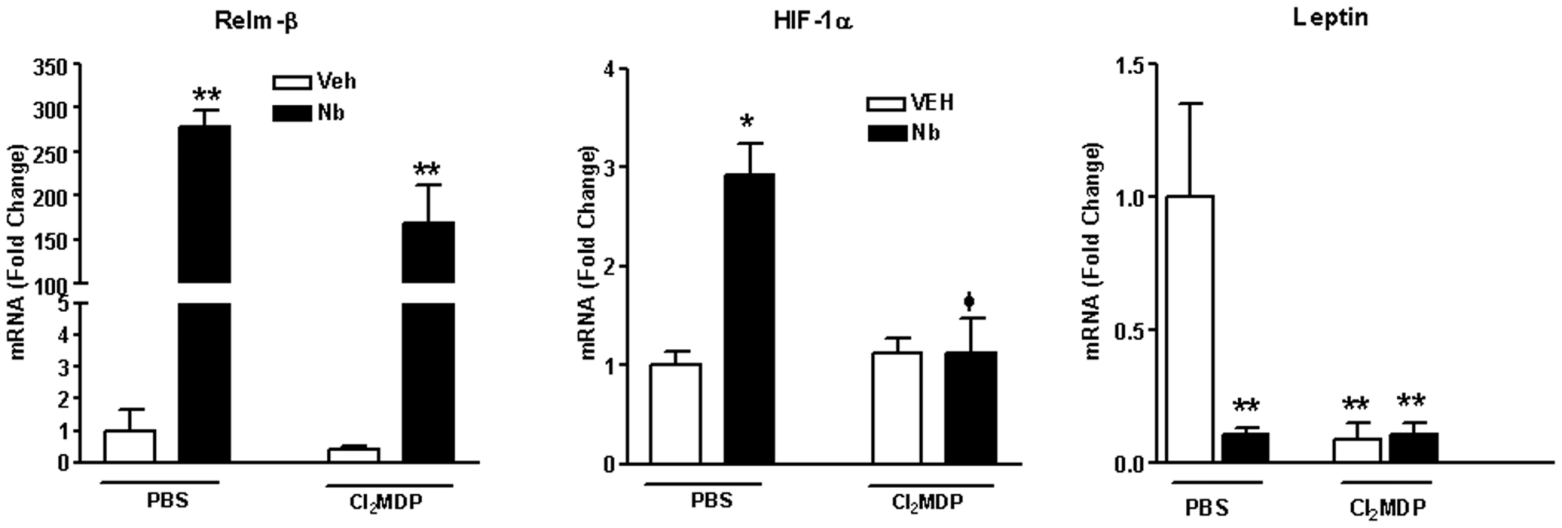
Macrophage depletion alters expression of factors involved in small intestinal glucose homeostasis. WT mice were given PBS or clodronate (Cl_2_MDP) containing liposomes to deplete macrophages and one day later treated with vehicle (VEH) or infected with *N. brasiliens*is (Nb). At day nine post-inoculation, full thickness sections of small intestine were prepared for real-time qPCR to determine the mRNA expression of RELM-β leptin, or HIF-1α. Values shown are means ± SE, *p<0.01 vs. VEH Control; φp<0.05 vs *N. brasiliensis*-infected mice treated with PBS-containing liposomes.

There are a number of factors increased by nematode infection that are associated with altered glucose metabolism. As shown previously [34], [35], RELM-β is increased significantly by nematode infection and is necessary for IL-4/IL-13-dependent expulsion of enteric nematodes (Figure 5A). The previously reported effects of RELM-β to down-regulate SGLT1 expression and enhance GLUT2-mediated transport of glucose [36] are inconsistent with the effects observed in this study. In addition, the increased expression of RELM-β in response to *N. brasiliensis* infection in the present study was independent of the presence of macrophages (Figure 5A).

The hormone leptin is produced primarily by adipocytes and has a number of functions relevant to glucose metabolism. It is produced also on both apical and basolateral surfaces of epithelial cells [37] where it inhibits SGLT1 activity *in vivo* [38] and up-regulates expression of GLUT2. Infection with *N. brasiliensis*, however, significantly decreased leptin expression (Figure 5B). Of interest is that there was also a reduction in leptin expression in mice treated with Cl_2_MDP alone suggesting that macrophages contribute to the control of leptin expression. Leptin expression, however, was not decreased further by *N. brasiliensis* infection in Cl_2_MDP-treated mice indicating that the down-regulation of leptin alone in response to nematode infection cannot explain the change in glucose transporters.

Hypoxia-inducible factor (HIF)-1α, a transcription factor in epithelial cells that mediates adaptive responses to changes in oxygenation, regulates the transcription of numerous genes known to be involved in barrier function as well as glucose and energy metabolism [39], [40]. Control of HIF-1α occurs primarily at the post-transcriptional level [41] and GLUT 1 is a major target gene of HIF-1α. HIF-1α is expressed by macrophages as well as epithelial cells [41]. The infection-induced elevation in HIF-1α expression was abolished completely in mice that received Cl_2_MDP identifying recruited macrophages as the source of newly expressed HIF-1α Figure 5C) The observed increase in the expression of HIF-1α-induced GLUT1 in captured (LCM) epithelial cells in response to infection can be attributed to the effects of HIF-1α in epithelial cells (Figure 4C).

## Discussion

Gastrointestinal helminth infection is associated with a decreased incidence of autoimmune diseases including inflammatory bowel disease (IBD) and diabetes. This concept was confirmed in experimental models using *Heligmosomoides bakeri* (formerly *Heligmosomoides polygyrus*) to prevent the development of colitis [42]–[44] as well as the onset of type-1 diabetes in non-obese diabetic (NOD) mice [3], [4]. The mechanism of these beneficial effects is an area of active investigation yet in each of these pathologies, innate immune cells such as macrophages play a key role in the inflammatory process. Among the infiltrating immune cells during infection, macrophages can manipulate the host both for immunologic and metabolic ends [45]. In the present study M2 play a key role in the parasitic nematode infection-induced changes in glucose transport by reducing the activity of the major intestinal glucose transporter, SGLT1. In addition, *N. brasiliensis* infection also induced macrophage-dependent and –independent alterations in the expression of other epithelial cell glucose transporters including GLUT1 and GLUT2 to alter dramatically the absorption of glucose into the enterocyte. Thus, parasitic infection results in a shift from insulin-dependent to non-insulin dependent transporters and magnifies the contribution of paracellular glucose absorption. These data demonstrate that several aspects of epithelial glucose absorption are regulated by immune driven mechanisms during parasitic infection.

Glucose is absorbed in the small intestine by transcellular pathways utilizing transporters as well as by paracellular pathways through solvent drag, a process that is modulated by changes in intestinal permeability. Our results demonstrate that M2 do not contribute to the increase in intestinal permeability during infection as decreased mucosal resistance was observed in infected mice irrespective of treatment with Cl_2_MDP. The increased permeability, however, likely enhances the contribution of paracellular absorption of glucose during nematode infection. This may help to maintain an adequate supply of glucose and contribute to the maintenance of body weight observed during infection in the present study. SGLT1 is one of the major transporters of glucose absorption following a meal, and is expressed constitutively at high levels in the small intestine and kidney [46]. Its activity is linked to the presence of carbohydrates and sodium as SGLT1 drives passive water absorption, a feature critical to the success of oral rehydration therapy. A previous study in *N. brasiliensis*-infected rats showed a decrease in SGLT1 protein expression in the jejunum, but not the ileum [15]. It should be noted that *N. brasiliensis* is a natural infection in rats and is associated with significant villus atrophy, epithelial apoptosis, and neutrophil infiltration. This parasite was adapted to infect mice and the intestinal pathology is much less severe in this species. The present study in mice shows that nematode infection reduced the activity, but not the expression, of SGLT1, and that M2 were critical for this decrease in SGLT1 activity. Of interest is that rotavirus infection also induces a specific inhibition of SGLT1 activity without changing gene or protein expression [47]. In both rotavirus and *N. brasiliensis* infection, the accumulation of intraluminal fluid occurs in the absence of significant pathology. In the present study, it is likely that macrophages influence glucose handling in epithelial cells much as they modulate glucose metabolism in adipocytes. Macrophages derive most of their metabolic energy from glucose, and may inhibit glucose uptake in other cells to satisfy their own energy demands. Thus, this is the first study to show that macrophages can impact glucose transport in epithelial cells.

The decrease in SGLT1 activity in the current study was associated with a reduction in GLUT2 gene and protein expression. GLUT2, a high capacity, low affinity, facilitative glucose transporter, was initially thought to be localized only to the basolateral membrane, however, it was later demonstrated that high concentrations of luminal glucose (>30 mM) induce translocation of GLUT2 to the apical membrane [48]–[50]. Trafficking of GLUT2 to the apical membrane is controlled in part by SGLT1 activity and provides a cooperative mechanism whereby glucose absorption can be matched to dietary intake. Apical GLUT2 is thought to be both a short term (high glucose meal) and long term (obesity) adaptation of intestinal epithelial cell function [51]. Stress has been shown to reduce SGLT1 activity [52] and to both increase [52] and decrease [53] GLUT2 expression. Both apical GLUT2 and GLUT5 expression are up-regulated in experimental models of diabetes [54] and in obesity [51] and this accumulation is associated with insulin resistance and hyperglycemia. Epithelial GLUT2 expression is lower in lean versus obese individuals and insulin promotes internalization of GLUT2 during fasting [48]. GLUT2 expression remains low in M2 depleted as well as STAT6^−/−^ mice infected with *N. brasiliensis*, showing a lack of immune regulation. These data suggest that in the present study nematode infection provides signals that lead to down-regulation of GLUT2 in enterocytes.

The non-immune mechanism of this effect on GLUT2 is unclear, but the factors that regulate GLUT2 expression and apical versus basolateral localization are an area of active research. There are several factors that are altered during nematode infection that also affect GLUT2 expression. Goblet cell secretion of high levels of RELM-β is critical to worm clearance [34], [35]. The expression of RELM-β, however, is associated with an increase in glucose absorption as a result of elevated SGLT1 activity and increased GLUT2 expression [36]. In the current study, RELM-β expression is up-regulated even in the absence of macrophages, indicating that RELM-β does not contribute to the observed macrophage-mediated changes in glucose handling during nematode infection. The hormone leptin has an inhibitory effect on appetite and is associated with increased epithelial GLUT2 expression [38], [55]. Importantly, leptin enhances Th1, but suppresses Th2 responses [56], [57]; therefore, it is not surprising that the upregulation of Th2 cytokines during nematode infection coincides with reduced leptin expression. These data in BALB/c mice are consistent also with other nematode resistant strains [58] that develop a strong type 2 immune response that protects against the metabolic demands associated with chronic infection. Together, these data provide further evidence of a link between metabolism and immune function, such that during infection the macrophage-dependent inhibition of epithelial SGLT1 activity and macrophage-independent inhibition of GLUT2 both contribute to the reduced capacity to absorb glucose.

Coincident with the decrease in SGLT1 activity and GLUT2 expression was a significant increase in GLUT1 expression, at both mRNA and protein levels. GLUT1 is a high affinity transporter, known to be expressed by macrophages [59], so it was not unexpected that infection, which enhances macrophage recruitment, resulted in an increase in expression of GLUT1 in total tissue. Our results show, however, that GLUT1 expression remained significantly elevated in *N. brasiliensis*-infected macrophage-depleted mice, albeit at lower levels than in infected mice treated with PBS containing liposomes. LCM studies further revealed that there was a major increase in the expression of GLUT1 in epithelial cells during infection when compared to the lamina propria, the site of macrophages. GLUT1 is a constitutive transporter that is insulin-independent. These data indicate that the upregulation of GLUT1 may be a compensatory response to the low glucose transport in the enterocyte during nematode infection. The present study shows that during nematode infection, epithelial expression of GLUT1 can be induced in the small intestine by the stress of infection, and may compensate for reduced activity of SGLT1 and down-regulation of GLUT2 expression.

Previous studies showed that alterations in glucose concentrations, as well as the inflammatory microenvironment, alter the expression and availability of HIF1α, a major transcription factor in the cellular response to hypoxia [60]. Of interest is that HIF-1α also regulates expression of genes important for glucose metabolism [61] including GLUT1 [62], [63]. In the present study, there was a significant reduction in the infection-induced up-regulation of HIF1α when mice were treated with Cl_2_MDP-loaded liposomes, indicating that the source of increase in transcripts was attributable to the influx of macrophages. Macrophages are unique in using glycolysis for ATP production constitutively rather than oxidative phosphorylation even during normoxia [64], explaining therefore that even M2 macrophages would exhibit high level of expression of HIF-1α. There is also a constitutive expression of HIF-1α in epithelial cells. Elevated glucose inhibits HIF-1α activation implying that in the present study, the inhibited SGLT1 activity and down-regulated expression of GLUT2 in epithelial cells results in low glucose levels and activation of HIF-1α This is consistent with the increased HIF-1α-induced GLUT1 expression in epithelial tumors to match increased energy demands [65]. The emerging role of HIF-1α in mucosal barrier function [66] may also be an important protective mechanisms in the face of the infection-induced increase in permeability.

**Figure 6:**
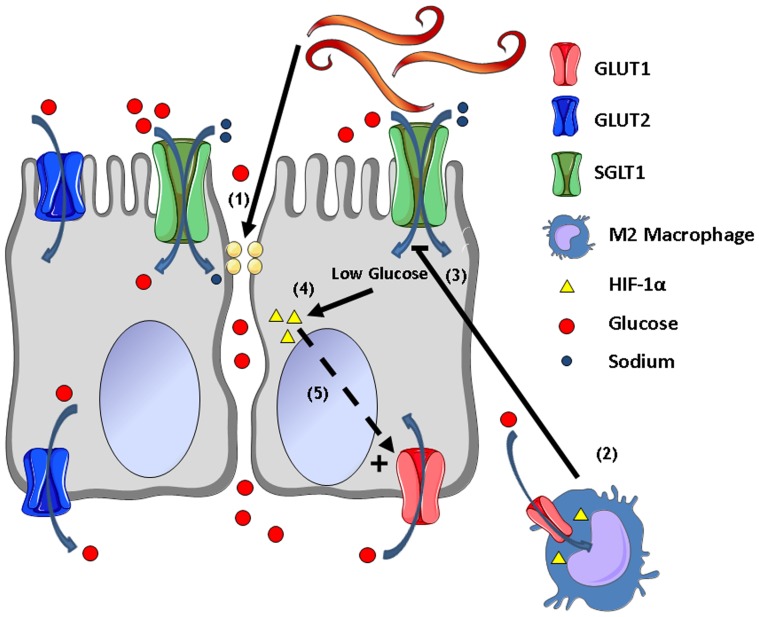
Model for changes in epithelial glucose handling in response to *N. brasiliensis* infection. (1) Infection induces a STAT6-dependent increase in mucosal permeability and (2) recruitment of alternatively activated macrophages (M2). (3) There is a M2-dependent inhibition of epithelial SGLT1 activity and M2-independent down-regulation of GLUT2. (4) This stresses the cell leading to activation of HIF-1α and up-regulation of the constitutive insulin-dependent GLUT1. This transporter serves as the major mechanism for glucose entry into the cell. Thus, parasitic nematode infection results in a “lean” epithelial phenotype as a result of a shift from insulin-dependent to insulin-independent glucose transporters. The increased permeability (1) enhances the contribution of paracellular absorption of glucose to maintain body weight during infection.

In conclusion, nematode infection induces both immune and non-immune changes in the function and expression of epithelial glucose transporters (Figure 6) including a macrophage-mediated decrease in SGLT1 activity and a non-immune down-regulation of GLUT2. The resulting low intracellular glucose in enterocytes is associated with a stress-related up-regulation of the less efficient and constitutive transporter GLUT1 as a major mechanism for glucose entry into the cell. These alterations are features of what can be termed the “lean” intestinal epithelial phenotype. The STAT6-dependent increase in intestinal permeability facilitates paracellular absorption of glucose to compensate for the reduced epithelial absorption. This may be linked to the “lean mouse phenotype” that expends energy on immune function to the detriment of energy stores [58]. These effects may contribute to the beneficial effects of nematode infection and type 2 cytokines against pro-inflammatory pathologies including diabetes and obesity.
